# Multiscale Modeling of Influenza A Virus Infection Supports the Development of Direct-Acting Antivirals

**DOI:** 10.1371/journal.pcbi.1003372

**Published:** 2013-11-21

**Authors:** Frank S. Heldt, Timo Frensing, Antje Pflugmacher, Robin Gröpler, Britta Peschel, Udo Reichl

**Affiliations:** 1 Max Planck Institute for Dynamics of Complex Technical Systems, Magdeburg, Germany; 2 Institute for Analysis and Numerics, Otto von Guericke University, Magdeburg, Germany; 3 Chair of Bioprocess Engineering, Otto von Guericke University, Magdeburg, Germany; Duke University, United States of America

## Abstract

Influenza A viruses are respiratory pathogens that cause seasonal epidemics with up to 500,000 deaths each year. Yet there are currently only two classes of antivirals licensed for treatment and drug-resistant strains are on the rise. A major challenge for the discovery of new anti-influenza agents is the identification of drug targets that efficiently interfere with viral replication. To support this step, we developed a multiscale model of influenza A virus infection which comprises both the intracellular level where the virus synthesizes its proteins, replicates its genome, and assembles new virions and the extracellular level where it spreads to new host cells. This integrated modeling approach recapitulates a wide range of experimental data across both scales including the time course of all three viral RNA species inside an infected cell and the infection dynamics in a cell population. It also allowed us to systematically study how interfering with specific steps of the viral life cycle affects virus production. We find that inhibitors of viral transcription, replication, protein synthesis, nuclear export, and assembly/release are most effective in decreasing virus titers whereas targeting virus entry primarily delays infection. In addition, our results suggest that for some antivirals therapy success strongly depends on the lifespan of infected cells and, thus, on the dynamics of virus-induced apoptosis or the host's immune response. Hence, the proposed model provides a systems-level understanding of influenza A virus infection and therapy as well as an ideal platform to include further levels of complexity toward a comprehensive description of infectious diseases.

## Author Summary

Influenza A viruses are contagious pathogens that cause an infection of the respiratory tract in humans, commonly referred to as flu. Each year seasonal epidemics occur with three to five million cases of severe illness and occasionally new strains can create pandemics like the 1918 Spanish Flu with a high mortality among infected individuals. Currently, there are only two classes of antivirals licensed for influenza treatment. Moreover, these compounds start to lose their effectiveness as drug-resistant strains emerge frequently. Here, we use a computational model of infection to reveal the steps of virus replication that are most susceptible to interference by drugs. Our analysis suggests that the enzyme which replicates the viral genetic code, and the processes involved in virus assembly and release are promising targets for new antivirals. We also highlight that some drugs can change the dynamics of virus replication toward a later but more sustained production. Thus, we demonstrate that modeling studies can be a tremendous asset to the development of antiviral drugs and treatment strategies.

## Introduction

Influenza A viruses continue to pose a serious threat to public health causing three to five million cases of severe illness and up to 500,000 deaths during the annual epidemics [1]. In addition, novel influenza strains that acquire the potential to infect and transmit efficiently between humans can create pandemics like the 1918 Spanish Flu that killed millions worldwide [2]. Currently, there are only two classes of direct-acting antivirals (DAAs) licensed for influenza treatment: fusion inhibitors (adamantanes), which impair virus entry, and neuraminidase blockers (oseltamivir and zanamivir) interfering with the release of progeny virus particles [3]. However, resistances against these drugs occur frequently [4], urging the need for new antiviral agents [6].

In recent years, the discovery of new antiviral targets for influenza treatment has received much attention. In particular, compounds which interfere with host factors promise to be effective antivirals as cellular factors are less susceptible to mutation impairing viral escape strategies. Such compounds can, for example, inhibit virus entry by removing cell surface receptors as was shown for recombinant sialidases, or block viral RNA transcription through PolII inhibition (for a detailed review of cellular targets and their inhibitors see reference [6]). The inhibition of essential cellular signaling cascades like Raf/MEK/ERK signaling, NF-κB signaling, the PI3K/Akt pathway, or the PKC signaling cascade is another promising strategy (reviewed in [7]). Finally, viral proteins themselves are targets for antiviral agents with new inhibitors of the viral neuraminidase, M2 ion-channel, and polymerase on the horizon (reviewed in [8]).

With the advent of these DAAs influenza therapy has moved beyond symptomatic treatment toward specifically targeting key steps of viral replication. The development of new and more potent drugs thus requires a deeper understanding of the viral life cycle [6]. In general, the growth of influenza viruses within a host involves at least two distinct scales: (i) the intracellular level of infection where the virus synthesizes its proteins, replicates its genome, and assembles new virions and (ii) the extracellular level at which it infects new target cells and spreads throughout the tissue. As DAAs can target both scales, understanding how these levels interact and where to interfere to efficiently counteract an infection is vital to the design of new antivirals.

In the past, mathematical modeling has provided valuable insights into the kinetics of influenza A virus infection under drug treatment ([9]–[13], reviewed in [14], [15]). However, the majority of studies focused exclusively on the extracellular level of infection either neglecting or drastically simplifying intracellular events. While such simplifications allow for the identification of critical infection parameters from sparse data, they can influence model predictions leading to an overly optimistic assessment of the treatment efficiency required to suppress the infection [16]. Other theoretical works examined how drugs affect specific replication steps of different viruses inside an infected cell [17]–[19]. Yet, these approaches only consider a single round of infection and do not account for the spread of the virus to new cells. Recently, Guedj and colleagues showed that combining both levels in a model of hepatitis C virus infection significantly improved its capability to explain clinical observations [20], [21]. However, as the authors only included viral genome copies at the intracellular level their approach is limited to the analysis of drugs that target genome synthesis, degradation or packaging. Nevertheless, such studies strongly suggest that integrating the intracellular life cycle of a virus into a model for cell-to-cell transmission would facilitate a systematic exploration of new drug targets. The resulting multiscale model can also yield a more realistic description of virus infection [22], [23] and more accurate estimates of key infection parameters [16], [20], [24].

Recently, we developed a model of the complete intracellular life cycle of influenza A virus comprising key steps from virus entry to progeny virion release [25]. Here, we link this description to the transmission of virus between host cells. We first show that this integrated modeling approach successfully captures data on the intracellular level of all three viral RNA species as well as on the extracellular infection dynamics represented by virus titers and the amount of infected cells. We then use the model to investigate potential antiviral targets including the steps of virus entry, nuclear trafficking, viral RNA and protein synthesis, and assembly/release. We provide a ranking of these targets and show that the lifespan of infected cells can be of particular importance for therapy success. Finally, detailed information on the construction of the model is provided in the Materials and Methods section at the end of this manuscript.

## Results

### Multiscale model of influenza virus infection

**Figure 1:**
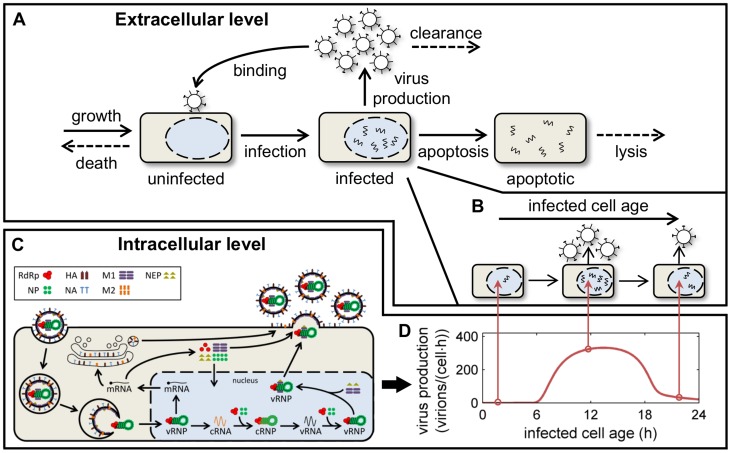
Schematic depiction of the multiscale model. (A) The extracellular level of infection comprises the growth and death of uninfected cells, their infection by free virions, the production of virus by infected cells, viral clearance/degradation, virus-induced apoptosis, and the lysis of apoptotic cells. (B) Infected cells are further segregated according to their infection age, i.e., the time that has elapsed since their infection. (C) The intracellular state of an infected cell is simulated using the model of influenza virus replication by Heldt *et al.* (see text and reference [25] for details). (D) Both levels are coupled via the age-dependent virus production rate, which depends directly on the internal state of a cell and determines the number of virions released into the extracellular space.

Our description of the extracellular level of infection is based on the classical model of viral kinetics within a host or cell population, which accounts for uninfected cells, infected cells, and free virions (reviewed in [14] and [15]). We augmented this framework by explicit consideration of the number of apoptotic cells and by modeling virus entry in more detail (Figure 1A). Once inside a cell, the virus starts producing viral RNA and proteins. To track these intracellular processes our multiscale model accounts for the age of an infected cell, i.e., the time that has elapsed since its infection (Figure 1B). The amount of each viral component inside an infected cell over its infection age is simulated using a model of the influenza A virus life cycle [25]. This submodel includes the following essential features of viral replication (Figure 1C): the production of viral mRNA and complementary RNA (cRNA) from viral ribonucleoproteins (vRNPs), which contain the negative-strand viral genomic RNA (vRNA); the synthesis of viral proteins; the encapsidation of newly produced cRNA and vRNA into cRNPs and vRNPs, respectively, by the viral RNA-dependent RNA polymerase (RdRp) and the nucleoprotein (NP); the nuclear export of vRNPs regulated by the viral matrix protein 1 (M1) and the nuclear export protein (NEP); and the assembly and release of progeny virions (for further details see reference [25]). Integration of both levels is achieved by assigning the age-dependent state of a cell to the age-segregated cell population (Figure 1D). In the model, intracellular replication primarily affects the extracellular level via the virus release rate, which depends on the abundance of viral proteins and RNA inside a cell and determines the amount of virions released into the extracellular space. The extracellular level in turn controls the number of infected cells and their lifespan.

### Viral kinetics in the absence of drugs

**Figure 2:**
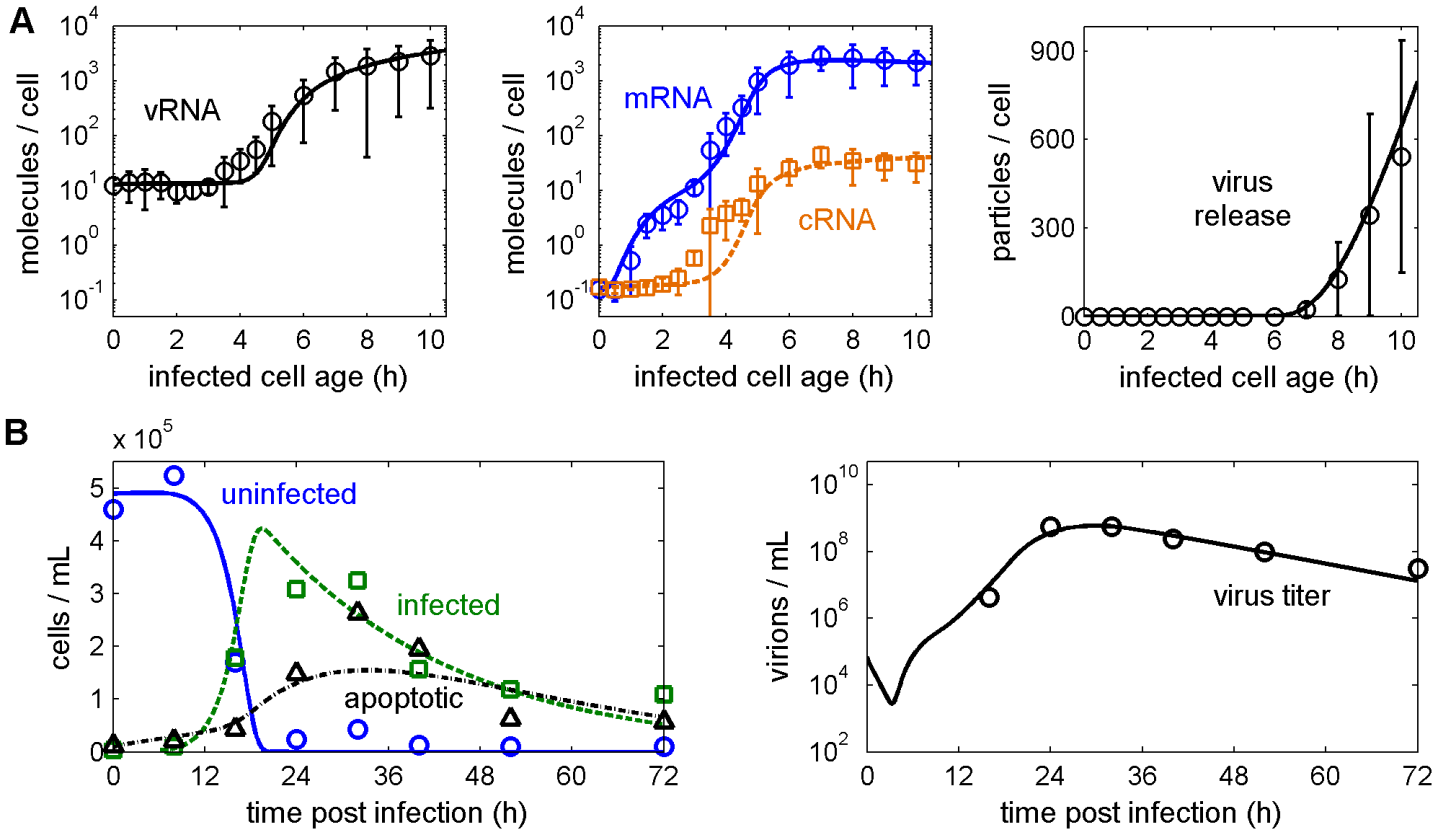
The multiscale model captures the intracellular and extracellular level of infection. Curves represent model fits to experimental infections of MDCK cells with influenza A/PR/8/34 (H1N1) depicted by symbols. (A) Levels of vRNA, cRNA (dashed, □) and mRNA (solid, ○) of segment 5 (encoding NP) and the amount of virus particles produced by an average infected cell in a synchronous, single round infection experiment (MOI = 6). Particle numbers correspond to the amount of hemagglutinating virus particles and were calculated from virus titer measurements by HA assay using Equation (12). Bars indicate the standard deviation of three independent experiments (two for the 9 and 10 hpi measurements). (B) Concentration of uninfected (solid, ○), infected (dashed, □) and apoptotic (dash-dotted, Δ) cells and infectious virus titer during multicycle infection (MOI = 0.1). Time courses were adopted from Isken *et al.* and are representative of three independent experiments [35].

To ensure an accurate calibration of both levels of the model, we followed a two-way strategy. First, we conducted experiments at a high multiplicity of infection (MOI), i.e., a high initial number of virions per cell, which results in a single synchronous infection round. This allowed us to measure the intracellular levels of the three viral RNA species together with the number of released virus particles and view them as the response of an average infected cell (Figure 2A). We then performed flow cytometry of low MOI experiments to assess the dynamics of multicycle infections where the virus spreads throughout a cell population in successive waves (Figure 2B). The model was fit simultaneously to both data sets such that the intracellular part, i.e., the replication inside an average infected cell agrees with the synchronous infection experiments, while its combination with the extracellular model captures the multicycle scenario. Hence, each infected cell behaves according to the time courses shown in Figure 2A and a population of such cells yields the dynamics in Figure 2B when infection occurs at low MOI.

Simulation results on both levels are in good agreement with the data showing, for instance, a rapid increase in viral mRNAs upon infection (Figure 2A). In contrast, cRNA and vRNA synthesis does not start until 3–4 h post infection (hpi) as the accumulation of viral proteins is required for genome replication [26]. Between 3 and 4.5 hpi our model underestimates the cRNA level. However, it does capture the amount of mRNA and vRNA. Since all three viral RNA species are tightly related the model is relatively constrained. Thus, some deviations are to be expected as the model has to balance these time courses as well as the data on the extracellular level. In the late phase of infection progeny vRNPs, which provide the template for cRNA and mRNA, leave the nucleus to be incorporated into new virus particles. This causes a shutdown of RNA synthesis around 5–6 hpi. At the same time, the first progeny virions leave the cell. Hence, the eclipse phase, i.e., the delay between infection and virus release, is approximately 6 h (Table 1). After this delay virus production increases as more viral components accumulate before it starts declining toward the end of the productive infection phase when proteins and later genome copies become limiting (Figure 1D). These intracellular dynamics fit well with the progression of infection on the extracellular level (Figure 2B) considering that typical errors of adherent cell numbers are in a range of 10–20% due to variations introduced by the measurement technique, handling and trypsinization. Most of the cells become infected between 12 and 19 hpi in the second and third wave of infection. Virus-induced apoptosis then causes a decline in cell numbers within the next two days. At 32 hpi, we observe a large discrepancy between the model and the data. However, this single time point can be regarded as an outlier since the measured total cell concentration increased by 30% between 24 and 32 hpi (data not shown). It is highly unlikely that such an increase occurs this late in infection. The good agreement of the model with the infectious virus titer provides further evidence for this. The titer shows an initial drop due to the attachment of seed virus to cells before it increases reaching its maximum around 30 hpi.

**Table 1:** Parameter estimates from data in Figure 2.

Parameter	Value	95% CI^a^
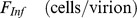	1^b^	0.47–1
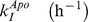	3.28×10^−2^	(2.26–5.90)×10^−2^
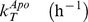	7.35×10^−3^	(4.89–11.09)×10^−3^
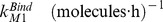	2.43×10^−4^	(0.59–4.35)×10^−4^
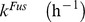	9.56×10^−3^	(3.95–21.31)×10^−3^
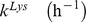	6.39×10^−2^	(4.64–8.83)×10^−2^
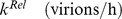	586	170–2650^c^
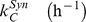	5.29	1.97–9.33
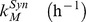 d	502	245–814
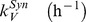	32.18	13.90–61.96
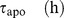 e	24.9	14.3–36.3
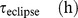 f	5.7	5.1–6.5

a 95% confidence intervals provided by the quantiles Q_0.025_ and Q_0.975_ of 2000 bootstrap replicates [56].

b One is the upper bound of this parameter as no more cells can become infected than virions fuse with endosomes.

c Estimates reached the lower and upper parameter bounds.

d Synthesis rate of an mRNA of average length. In the model, transcription is length dependent with a rate of 8.53×10^5^ nucleotides/h (see reference [25] for details).

e The average lifespan of an infected cell was calculated as 
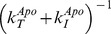
, which includes the eclipse phase.

f The end of the eclipse phase was defined as the time when the virus release rate 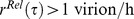. Note that this includes the steps of virus entry. The delay between fusion and virus release is only 2 h.

**Figure 3:**
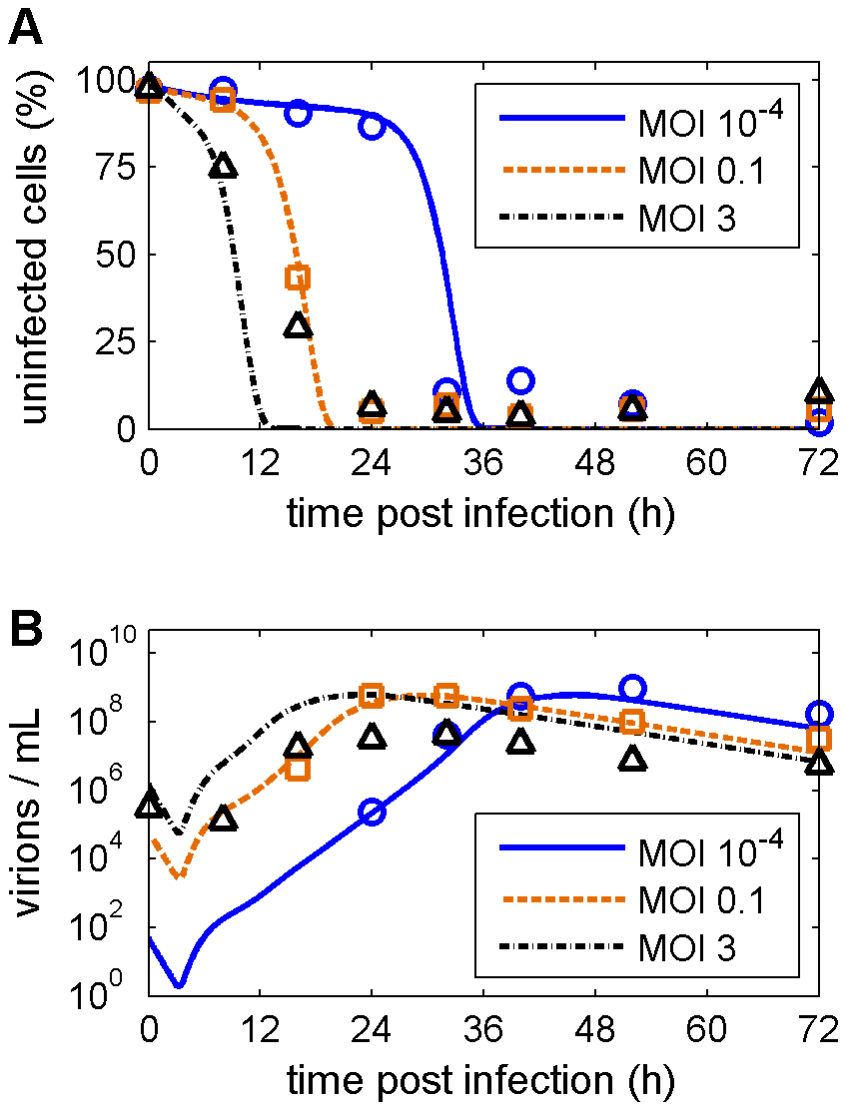
Model predictions reproduce data for different infection conditions. The model fit from Figure 2 (dashed, □) was used to predict the percentage of uninfected cells (A) and the infectious virus titer (B) for infections at an MOI of 10^−4^ (solid, ○) and of 3 (dash-dotted, Δ), respectively. These predictions were compared to data sets not used for model construction. Measurements were adopted from Isken *et al.* and are representative of three independent experiments [35].

Next, we checked the predictive capabilities of the model by comparing it to measurements at different infection conditions that were not used for model construction. These simulations successfully capture the shift of infection dynamics in the presence of higher and lower amounts of virus particles in the inoculum (Figure 3A). In addition, the virus titer prediction for a low seed virus concentration is in good agreement with experiments whereas for higher MOI virus production is overestimated (Figure 3B). We conclude that the model is in good agreement with the intracellular and extracellular dynamics of influenza A virus infection and can be of predictive value especially for low MOI regimes where multiple infection rounds occur, which resembles the *in vivo* situation more closely than single round experiments that use high MOI.

**Figure 4:**
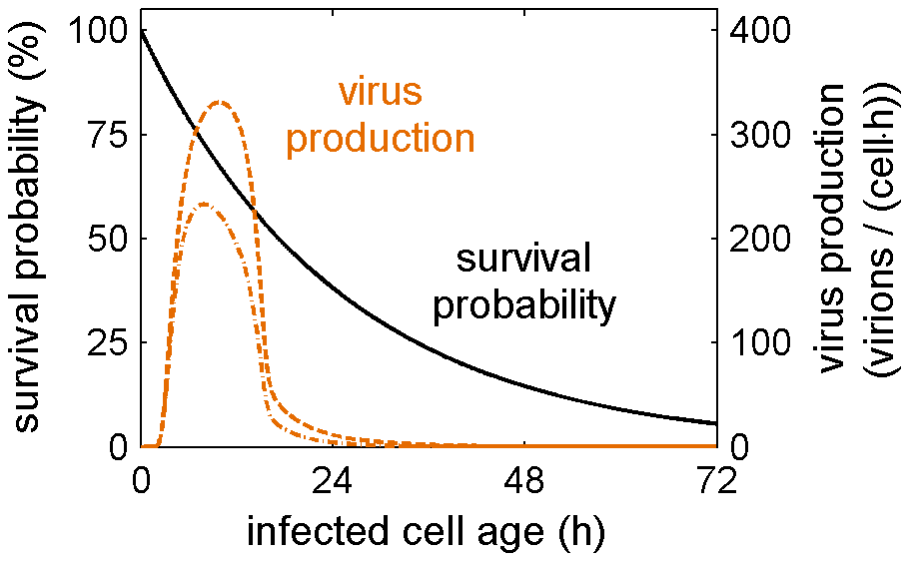
Cell death constrains virus production. Survival probability of an infected cell (solid) and virus production rate over the infected cell age neglecting cell death (dashed) and considering cell death (dash-dotted).

A major advantage of the proposed multiscale model is that it integrates the time course of intracellular virus replication with cell death dynamics. This allows us to assess whether the lifespan of an infected cell constrains virus production. From the measurements in Figure 2B, we obtain the average lifespan of an infected cell as 25 h (Table 1). Approximately at the same time virus release would stop due to the depletion of viral components (Figure 1D). Nevertheless, many cells may die before the end of this productive phase dependent on how much individual survival times vary around the mean. Models of viral infection usually assume that the probability of cell death is independent of time, i.e., that survival times follow an exponential distribution (see references [24] for more details and alternatives). Using this assumption, we find that most cells indeed die within 25 h with more than one quarter succumbing to apoptosis before reaching the peak in virus release (Figure 4). Hence, cell death can affect the number of virus particles an average cell produces.

### Effects of drug treatment

**Figure 5:**
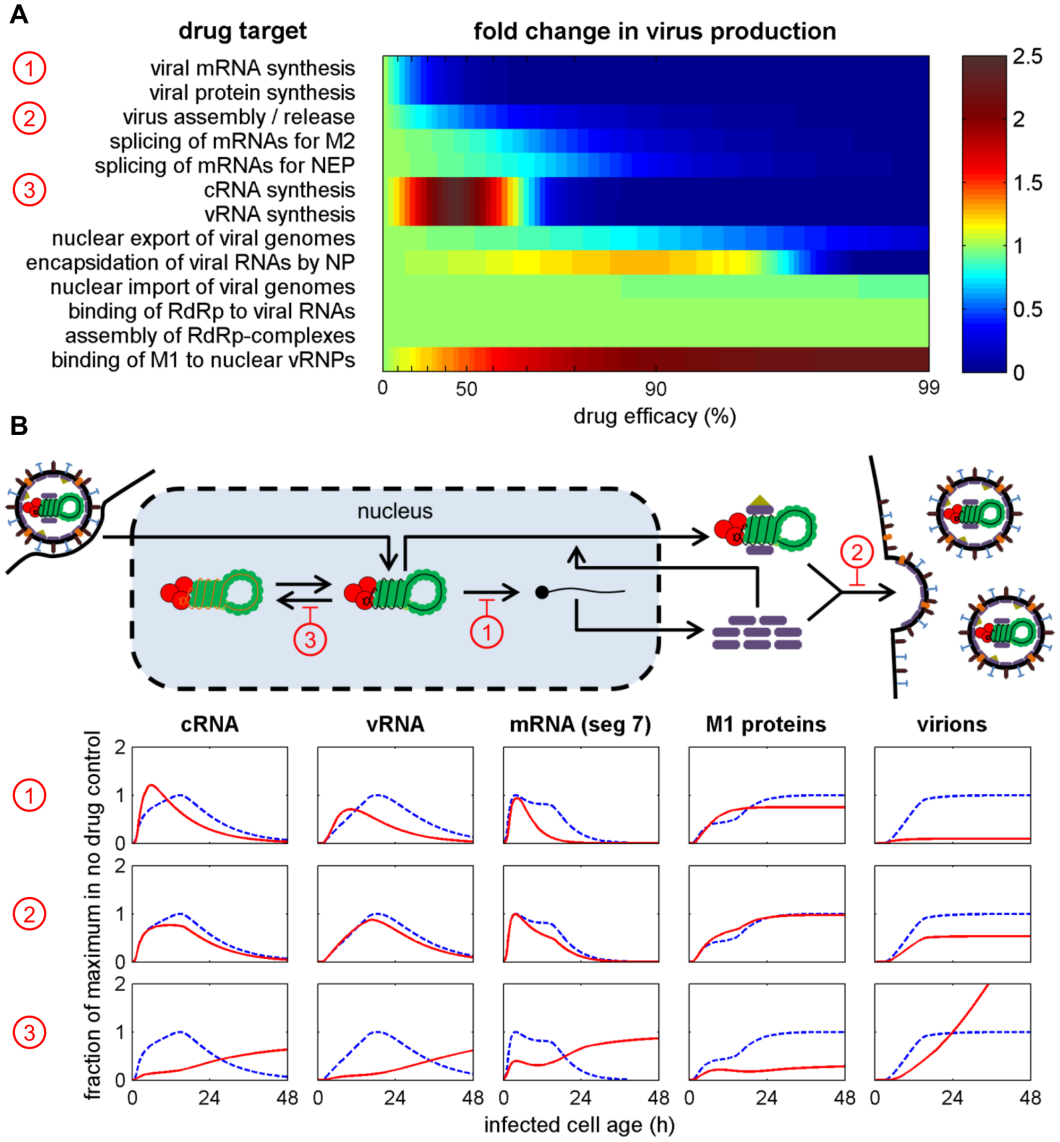
Inhibition of viral RNA synthesis, translation, or assembly/release efficiently impairs virus production. (A) Simulated impact of drugs targeting the indicated steps of intracellular virus replication with varying efficacy. Colors indicate the fold change in the total number of virus particles an average infected cell produces over its lifetime compared to the drug-free regime. Numbers in circles correspond to the examples shown in B. (B) Time courses of selected viral components during drug treatment with 50% efficacy. Columns correspond to components depicted in the scheme. Dashed and solid lines are time courses in the absence and presence of drugs, respectively. All components were normalized to their maximum in the drug-free regime.

Models of viral infection can be used to simulate the efficiency of antiviral treatment. While previous studies have mostly considered general effects of drugs on cell infection or virus production [9], [11], [14], our multiscale approach can also predict how drug interference affects the intracellular viral life cycle. Figure 5A shows simulation results that illustrate the impact of DAAs on the amount of virus particles an infected cell releases. In our model, inhibitors of viral mRNA synthesis, protein translation, and virus assembly/release are highly effective in reducing virus production even at low drug efficacy. Note that antivirals usually show a maximum efficacy above 90% [9], [10], [27]. At these levels, inhibitors of mRNA splicing, cRNA/vRNA synthesis, and nuclear export are also very successful. Intriguingly, inhibition of the two steps of RNA replication can, however, lead to an increase in virus release for low-efficacy drugs. A similar result can be observed when targeting M1 binding and the encapsidation of viral RNAs by NP. Figure 5B shows the simulated time courses of different viral components in response to selected low-efficacy drugs. As expected, the inhibition of viral transcription leads to lower mRNA levels, which impairs protein synthesis and virus release (Figure 5B upper panel). We also observe a minor increase in cRNAs and vRNAs during the early phase of infection due to lower M1 protein levels. Based on experimental evidence [28]–[30], M1 acts as a negative regulator of cRNA and mRNA synthesis in our model [25]. Together with NEP, it binds to vRNPs controlling their nuclear export. Once outside the nucleus, vRNPs can no longer serve as templates for the two positive-strand RNAs. In addition, M1 proteins fulfill a second role during virus assembly where they form the inner hull of virus particles as their most abundant viral component [31]. Hence, inhibition of particle assembly/release also results in higher M1 levels, a stronger negative regulation of RNA synthesis, and lower RNA levels besides reducing virus release (Figure 5B middle panel). This type of regulation also causes the increase in virus titers seen upon weak inhibition of cRNA synthesis (Figure 5B lower panel). The reduction in RNA levels in the early phase of infection leads to a lower abundance of M1 proteins. The resulting lack of inhibition allows a faster synthesis of RNAs during later stages and consequently a higher rate of virus release. Since the release of virions further drains the pool of M1 proteins, our model predicts a sustained production of virus particles for these drug efficacies.

**Figure 6:**
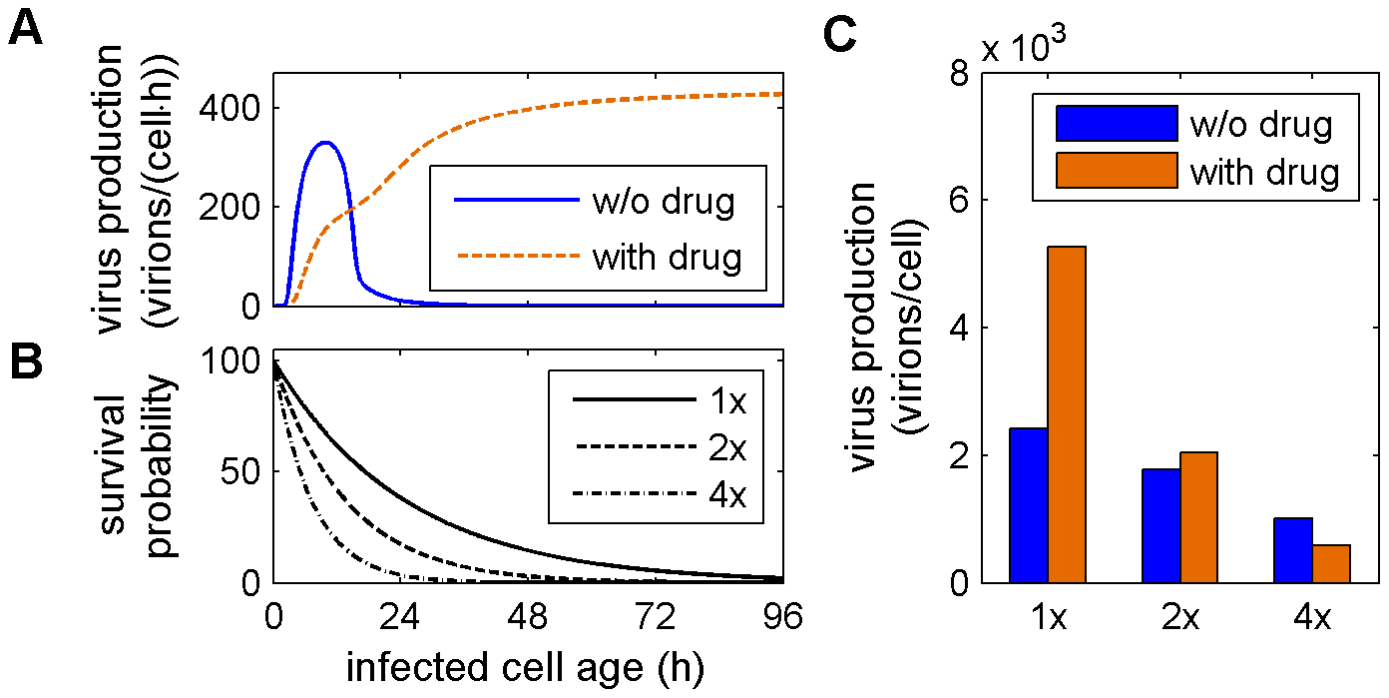
Cell death affects therapy success. (A) Virus production rate over the age of an infected cell in the absence of drugs (solid) and during inhibition of cRNA synthesis with 50% efficacy (dashed). (B) Different survival probabilities for an infected cell assuming that virus-induced apoptosis occurs with the rate estimated from data in Figure 2B (1×, solid line) or at twofold (2×, dashed line) and fourfold (4×, dash-dotted line) its rate. (C) Total amount of virus particles released by an infected cell considering the combination of different production rates and survival probabilities.

Figure 5B highlights an interesting aspect of viral replication. There may be regimes where a higher overall number of viruses can be produced at the expense of an early virus release. Yet, such an advantage would clearly depend on the lifespan of an infected cell and on whether cell death by virus-induced apoptosis or the immune response shortens it (Figure 6). While the inhibition of cRNA synthesis may lead to higher titers in our system, drug treatment has hardly any influence on virus production when apoptosis occurs at twofold of its estimated rate (Figure 6C 1×–2×). For an even shorter lifespan of infected cells the antiviral may be deemed effective reducing particle production to half its pre-treatment level (Figure 6C 4×). Hence, the effect of a drug on viral replication has to be judged with respect to cell death dynamics to correctly evaluate treatment potential.

**Figure 7:**
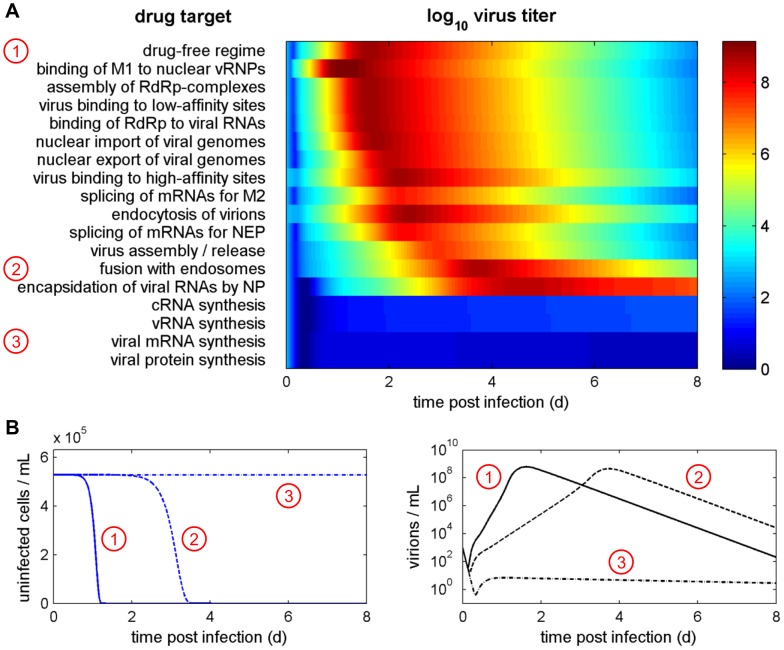
Inhibition of virus entry delays infection. (A) Simulated effect of drugs targeting the indicated steps of virus infection with an efficacy of 95%. Colors indicate the log_10_ virus titer. Numbers in circles correspond to the examples shown in B. (B) Concentration of uninfected target cells and virus titer in the absence of drugs (solid line) and during treatment with inhibitors of virus fusion (dashed) and mRNA synthesis (dash-dotted) at 95% efficacy.

Apart from reducing particle production, antivirals can also delay the spread of the virus providing time for the immune system to counteract infection. Figure 7A illustrates the evolution of virus titers in a susceptible host cell population under simulated drug treatment. Again, inhibitors of viral RNA and protein synthesis almost completely suppress viral replication. Therefore, these drugs protect most of the host cells from infection (Figure 7B). Nevertheless, a few cells become infected and produce virus causing the titer to only slowly decline. In contrast, drugs targeting virus entry, i.e., fusion, endocytosis and binding to the cell surface, are less successful in decreasing peak titers but delay infection by up to 50 h. However, they do not prevent the cell population from becoming infected. Note that, in this scenario, peak titers are primarily constrained by the number of available cells. When cells are depleted virus production ceases and titers decrease with the rate of viral clearance.

## Discussion

Viral infections cover several scales, a form of complexity that computational modeling is poised to address [32]. In this study, we developed a model that integrates the main stages of influenza A virus infection within a host: the intracellular replication of the virus and its extracellular transmission to new host cells. This multiscale approach accurately captures a variety of *in vitro* measurements, provides insights into virus growth across different scales, and aides the development of DAAs.

The limited quantity and diversity of experimental data still represents a significant bottleneck for models of extracellular viral kinetics and their validation [15]. A promising approach to close this gap is to incorporate detailed information on the intracellular viral life cycle. Our model of virus replication was previously validated against a variety of experimental studies including data on virus binding, fusion, RNA synthesis, and regulation by viral proteins [25]. In combination with the quantitative RNA levels presented here, it provides a detailed picture of intracellular events and their impact on virus production. For instance, it yields a delay of 6 h between infection and virus release and suggests that virus production increases for another 7 h as viral components accumulate. While the length of the eclipse phase is in good agreement with estimates from other modeling studies (7 h for MDCK cells in bioreactors [33], 0.22–6 h for cultivations in a hollow-fiber system [11] and 6 h for human infection [9]), previous models have assumed that virus production proceeds at a constant rate in the productive phase. Since simulations are quite sensitive to such assumptions [16], [24], multiscale modeling can lead to more realistic estimates of key infection parameters [20], which were shown to greatly support the design of antiviral treatment [34].

We estimate the lifespan of an infected cell as 25 h (including the eclipse phase), which is in the range of other studies (reviewed in [15]). Virus production would cease around the same time due to the depletion of viral components in the cell. Nevertheless, most of the cells in our simulations die before the end of the productive phase as survival times vary significantly around the mean. Following the majority of models for viral infection, we assumed an exponential distribution of the survival time. Yet, a recent study suggested that other distributions may be more appropriate to capture viral kinetics [24]. In principle, our model is ideal to accommodate such assumptions since the apoptosis rate can be chosen freely as a function of the infected cell age. However, our simulations (Figure 2) and data on apoptosis induction during single-cycle infections [35] do not justify more complex approaches.

Although in good agreement with the data, our simulations underestimate the intracellular level of cRNA between 3 and 4.5 hpi. Yet, both the mRNA and vRNA level are captured nicely. This might be due to a differential regulation of viral transcription and replication unaccounted for in the model so far. Such control could, for instance, be mediated by NP (reviewed in [36]), NEP [37] or small viral RNAs [38]. However, the contribution of these mechanisms is still a matter of controversial debate and quantitative data is lacking preventing us from incorporating this type of regulation in the model. We, thus, chose to keep our mathematical framework as simple and constrained as possible. Nevertheless, the model simultaneously captures a rich pool of data indicating that it incorporates the key steps of *in vitro* influenza virus infection.

When testing the model against data for different infection conditions that were not used for construction, we also noticed an overestimation of virus production for high MOI. In our model, peak virus titers primarily depend on the initial cell concentration, which was comparable in the three experiments and the number of virions each cell produces. The cell-specific virus yield follows from the intracellular replication dynamics and the lifespan of an infected cell (i.e. the yield equals the integral under the dash-dotted virus production curve in Figure 4). Hence, the observed decrease in virus production at high MOI can be explained in two ways. On the one hand, factors present in the inoculum may impair intracellular virus replication reducing the rate of virus production. Defective interfering particles could be such factors [39]. On the other hand, the inoculum can contain substances such as interferons that may reduce the lifespan of an infected cell by increasing apoptosis induction [35]. Experimental work is in progress to discriminate between these two hypotheses.

For the construction and calibration of our model, we mostly relied on cell culture experiments due to the limited diversity of available *in vivo* data. Currently, virus titers are the type of data most frequently used for *in vivo* models (reviewed in [14]) as they are easily attainable from infected individuals and animals. In principle, four parameters are sufficient to describe such titer curves constraining the level of detail one can incorporate into a mathematical model [24]. In contrast, *in vitro* systems provide access to a variety of information like the number of available cells, their infection status or the intracellular level of viral RNAs. This wealth of data was a prerequisite for the development of our multiscale model. However, now that the model has been established future studies may want to implement modifications to closer resemble the *in vivo* situation. For instance, the growth and death of uninfected cells is usually neglected in acute infection models as target cell dynamics are assumed to be slow compared to infection [14]. Furthermore, virus loss in the lung is caused by active processes such as phagocytosis and mucociliary clearance as opposed to degradation in cell culture experiments and may, hence, be faster. However, the most prominent feature of *in vivo* infections our model is currently lacking is the immune system. Although a number of models were proposed that incorporate an immune response none of them agreed completely with the variety of experimental data available [40]. Implementing an adequate description of the immune response, thus, remains one of the major challenges in viral kinetic modeling today [14], [40]–[42].

Modeling intracellular replication in detail allowed us to simulate the effect of DAAs on the amount of virions an average infected cell produces. Given a drug efficacy above 90% inhibitors of viral transcription, replication, protein synthesis, nuclear export, and assembly/release proved to be most successful in mitigating replication. Indeed, antivirals targeting virus release in the form of neuraminidase inhibition are widely used in influenza treatment today. To exploit this target in the future new compounds are, however, required as the emergence of drug-resistant strains is on the rise [8]. Inhibitors of the viral polymerase are a promising alternative. During viral replication polymerases engage in an autocatalytic reaction where they synthesize cRNA from vRNA and vice versa. In addition, they transcribe the viral genome into mRNAs for new polymerases. Interrupting this positive feedback has detrimental consequences for all major viral components in our model. In agreement with this, compounds which specifically inhibit viral transcription efficiently impair influenza A virus replication in cell culture and mice [43]. Similarly, favipiravir (T-705), an inhibitor of influenza virus RNA polymerase activity [44], is potent against influenza viruses *in vitro* and *in vivo* [45], [46] and has entered clinical trials recently [47] demonstrating the potential of viral RdRps as drug targets. Our model could be used to test different dosing regimes and support such clinical trials.

In other studies, interference with the assembly of viral polymerase complexes by 25-amino-acid peptides [48] or small molecule inhibitors [49] has been shown to inhibit viral replication. In contrast, RdRp formation has hardly any influence on virus production in our model unless the drug efficacy well exceeds 99%. This discrepancy most likely originates from the kinetics of polymerase assembly in our model. Due to the lack of quantitative data, we assumed that polymerases form from their three subunits according to mass actions kinetics [25]. Rather than the formation itself subunit availability represented the kinetic bottleneck for RdRp assembly in simulations. In light of the above mentioned experimental studies, future models may need to revise this assumption if polymerase assembly is at the focus of investigation. Reconciling model predictions that are initially inconsistent with data provides an ideal opportunity to also refine our understanding of the underlying biology but it requires experiments specifically designed to resolve the discrepancy.

Instead of reducing peak virus titers, our model predicts that inhibitors of virus entry mainly delay *in vitro* infection, which is in agreement with previous studies [11]. This is because they only decrease the infection rate of cells instead of impairing the processes responsible for viral component production resulting in similar cell-specific virus yields. The treatment success of such inhibitors may, hence, depend on mechanisms, which take advantage of the delay and clear infection.

Intriguingly, some of our simulations yield regimes where treatment can also lead to an increase in virus production at the expense of early virus release. From an evolutionary perspective this regime might not be beneficial as faster growing strains would out-compete such variants. However, during treatment it may, nevertheless, occur. We show that the lifespan of an infected cell determines whether a slower but more efficient virus production leads to higher titers. An antiviral treatment that was rejected based on the survival times of infected cells in cell culture may thus even be successful when lifespans are shorter. *In vivo*, the latter is indeed very likely as the immune response increases cell death rates [42], [50]. Also, virus strain-dependent factors such as the expression of the PB1-F2 protein can lead to faster cell death [51]. Screening approaches for antiviral compounds may, hence, benefit from using conditions that mimic the cell survival times observed *in vivo*.

In summary, we have developed a multiscale model of *in vitro* influenza A virus infection which integrates the intracellular level of viral replication and the extracellular level of cell-to-cell transmission. We are optimistic that such models will contribute to the development of antiviral drugs, support clinical trials and provide a platform for the establishment of more detailed infection models in the future. To achieve this goal, next-generation models will need to incorporate the immune response, pharmacokinetics and comprehensive information on virus-host interactions. Multiscale modeling provides an ideal framework for such an endeavor as diverse cellular processes can be simulated individually and incorporated as separate modules into a unifying framework.

## Methods

### Model of the extracellular level

We used an age-segregated infection model for adherent cells, which follows from the general population balance [23], to describe the dynamics of uninfected target cells (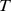), infected cells (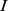), and their apoptotic counterparts 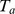 and 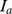, respectively
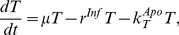
(1)

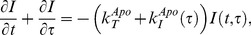
(2)

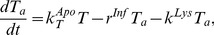
(3)

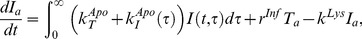
(4)with
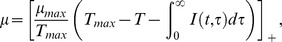
where uninfected cells grow with specific rate 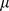 or undergo apoptosis with rate 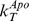. Growth can occur with a maximum specific rate 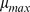 to a maximum concentration of 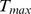
cells assuming that all non-apoptotic cells occupy a finite surface area. The infection rate is denoted 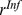 and will be discussed at the end of this section. In Equation (2), infected cells have the age 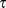 and undergo virus-induced apoptosis with an age-dependent rate 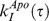. Since infection creates cells with age zero, we obtained the boundary condition 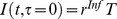. Apoptotic target cells in Equation (3) can either become infected or undergo cell lysis with rate 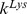. The same lysis rate is used for apoptotic infected cells.

Assuming that there are no infected cells in the beginning (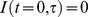), we can rewrite Equation (2) in terms of an algebraic equation
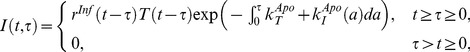
(5)where 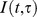 can be interpreted as the infection age density such that 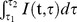 gives the number of infected cells with age between 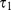 and 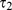. Equation (5) illustrates that cells which have age 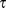 at time 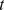 were infected at time 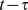. The integral term accounts for cell loss due to apoptosis. Using Equation (5) instead of Equation (2), thus, allows us to track the infection front precisely.

The equation for infectious virus particles (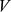) in the extracellular space follows as
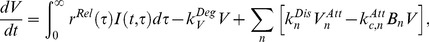
(6)with
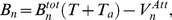


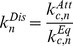
and
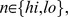
where 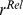 denotes the age-dependent virus production rate. We assumed that virions are degraded or cleared with rate 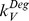. The binding of virus particles to target cells was modeled as described before [25]. In brief, we considered two types of binding sites (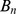): high-affinity (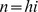) and low-affinity (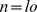) sites. The virus attaches to or dissociates from these sites with rates 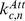 and 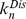, respectively, whereby the latter rate follows from the equilibrium constant 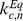. The concentration of free binding sites was calculated from their total number per cell (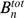), the concentration of target cells, and the concentration of attached virus particles (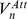). In this notation each virion occupies one binding site. Note that we did not consider binding to infected cells as neuraminidase expression on the cell surface limits superinfection [52].

In order to account for drug effects on virus entry, we defined equations for the concentration of attached virions (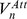) on the surface of target cells (considering both 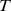 and 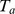) as well as for virions in the endosomes of these cells (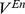)
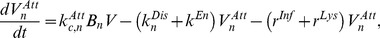
(7)

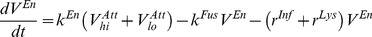
(8)where 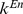 and 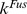 denote the endocytosis and fusion rate, respectively. The first two terms in Equation (7) account for virus binding and dissociation as well as for endocytosis. The last term quantifies the loss of virions with cells that leave the compartment of interest, i.e., with cells leaving the population of target cells by infection or cell lysis with rate 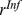 and 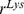, respectively. Equation (8) accounts for the endocytosis of virions attached to both types of binding sites, the fusion of virions with the endosomal membrane, and again the loss of particles due to infection and lysis of target cells.

Since we consider a cell ‘infected’ as soon as viral genome copies enter its cytoplasm, the infection rate 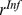 follows from the fusion rate in Equation (8)

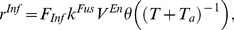
(9)with
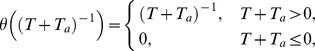
where 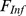 corresponds to the number of cells which become productively infected upon the fusion of one virion. This number cannot exceed one but may become lower if several virions are required to cause productive infection. While the first part of Equation (9) represents the number of cells that become infected per hour, the fraction serves two purposes: substituted in Equations (7) and (8) it provides the number of viruses per target cell and in Equations (1) and (3) it yields the fraction of non-apoptotic and apoptotic target cells, respectively, to total target cells. Similarly, the lysis rate of apoptotic target cells 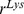 can be derived as
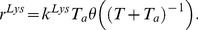
(10)


### Model of the intracellular level

The intracellular level of infection was essentially modeled as described before [25]. In brief, a set of ordinary differential equations was used to simulate virus entry, viral RNA and protein synthesis, and virus assembly. In contrast to the original description, we modified the equation of the virus release rate
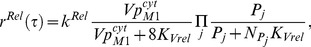
(11)with
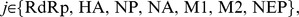
where release depends on the abundance of progeny vRNPs in the cytoplasm (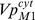) and structural viral proteins (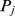) with 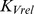 denoting the number of virus particles for which components must be present in order to reach half the maximum release rate. In its new form, 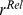 can only increase to a maximum rate of 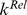 assuming that there is only a limited number of host factors available for virus budding. This change was implemented to avoid unrealistically high virus production rates that occurred in some treatment regimes.

For simulations in Figure 1D and Figure 2A, the complete intracellular model was used as described above. However, when coupling the model to the extracellular level, we neglected virus entry and initialized the model with a complete set of eight vRNPs in the cytoplasm. Attachment, endocytosis, and fusion were considered at the extracellular level instead (Equations (7) and (8)).

### Integrated simulation approach

In order to ease the computational burden and allow for a more intuitive interpretation of simulation results, we assumed that the extracellular level has little or no influence on intracellular events, i.e., that each infected cell behaves the same independent of the time of infection and the extracellular environment. As shown by Haseltine and colleagues, this assumption permits the selective decoupling of both levels and reduces the model's complexity significantly [23]. Hence, we could first simulate intracellular virus replication to calculate the virus release rate 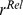 as a function of the infection age 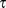. This rate was then used in Equation (6) to simulate the extracellular level.

The intracellular submodel was solved numerically with the CVODE routine from SUNDIALS [53] on a Linux-based system. Model files and experiments were handled with the Systems Biology Toolbox 2 [54] for MATLAB (R2010b The MathWorks Inc.). We then used Euler's method with a step size of 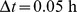 to solve the extracellular model (Equations (1) and (3)–(8)). The integrals in Equations (1), (4) and (6) were approximated in each step by substituting Equation (5) for 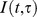 and using the rectangle rule with a step size of 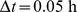. To further reduce computational costs, the integral in Equation (5) was evaluated prior to simulation following the same approach. The method was checked for numerical accuracy against simulations using smaller step sizes and by comparison to a discrete version of Equation (2) with a large number of age classes.

Table S1 lists the initial conditions of all presented simulations. Parameters of the intracellular model can be found in Table S2 and Table S3 shows parameter values for the extracellular model.

### Parameter estimation

Model parameters were estimated by fitting the complete intracellular submodel (including the equations for virus entry) to experimental virus titers per cell and the levels of vRNA, cRNA and mRNA measured during high MOI infection (Figure 2A). Simultaneously, the reduced model (excluding virus entry) was coupled to the extracellular equations using the same parameters and the complete multiscale model was fit to the time courses of uninfected and infected cells, their apoptotic counterparts, and the virus titer during low MOI infection (Figure 2B). Estimation was performed using the fSSm algorithm for stochastic global optimization [55]. In particular, the algorithm was used to simultaneously minimize the least squares prediction error of all measured state variables, whereby the error of each variable was normalized by its respective maximum measurement value (e.g. the deviation between measured and simulated vRNA level was weighted by the maximum of the measured vRNA level). The summed errors of the intracellular and extracellular part of the model were then divided by the number of measurements, respectively, and added to attain an overall measure of fit quality. Since experiments indicated that real-time RT-qPCR detects free viral RNAs from the seed virus supernatant, which may adhere to cells but cannot enter them, we applied the first measurement value as an offset to all simulation values of viral RNAs. Bootstrap confidence intervals [56] were determined considering the standard deviations in Figure 2A as well as a 20% error for cell counts and 0.3 log for virus titers in Figure 2B.

### Simulation of drug treatment

In order to simulate drug treatment with efficacy 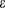, parameters in the model which correspond to the drug's target (Table S4) were perturbed by 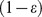. Treatment was assumed to occur at constant efficacy starting from 0 hpi. For results in Figure 5A, the reduced intracellular model was simulated first to determine the virus release rate 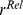. The total amount of virus particles produced by an average infected cell over its lifetime (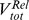) was then calculated by considering cell death according to
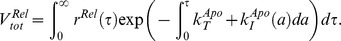



### Cell culture and virus infection

For single round infections (Figure 2A), adherent MDCK cells (ECACC No. 84121903) were grown in GMEM (GIBCO) supplemented with 10% fetal calf serum (FCS) (PAN Biotech) and 1% peptone (Lab M) using T175 flasks and incubated at 37°C under a 5% CO_2_ atmosphere to maintain pH 7.2. One day before infection, cells were washed twice with phosphate buffered saline (PBS), detached and counted with a Vi-CELL XR (Beckman Coulter). Subsequently, 1.75×10^6^ cells were seeded into 35 mm dishes. Infection was performed using influenza A/Puerto Rico/8/34 (Robert Koch Institute, #3138) with a seed virus preparation containing 1.23×10^8^ infectious virus particles per mL. Prior to infection, cells were washed twice with PBS and virus was added at a multiplicity of infection (MOI) of 6 in 250 µL serum-free virus maintenance medium (GMEM, GIBCO) containing 1% peptone (Lab M) and 5 units/mL trypsin (GIBCO). Dishes were incubated for 30 minutes at 37°C and 5% CO_2_ atmosphere before cells were washed once with PBS and 1 mL virus maintenance medium was added.

To correctly account for the loss of viral components due to virus release, the total amount of virus particles leaving an average infected cell was determined using the hemagglutination assay as described previously by Kalbfuss et al. [57]. Titer measurements in log_10_ HA units per test volume (log HAU/100 µL) can be converted into hemagglutinating particles per mL by
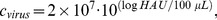
(12)assuming that at least one virus particle per erythrocyte (2×10^7^ cells/mL) is required to cause agglutination [58].

For detailed information on the multicycle experiment (Figure 2B), the reader is referred to reference [35] from which the measurements were adopted. In brief, adherent MDCK cells were cultivated to confluence in T25-flasks and washed with PBS prior to infection followed by addition of serum-free virus maintenance medium (GMEM, GIBCO) containing 1% peptone (Lab M) and 5 units/mL trypsin (GIBCO). Subsequently, influenza A/Puerto Rico/8/34 was added at an MOI of 10^−4^, 0.1 and 3. For each time point one T-flask was harvested and adherent cells were trypsinized and pooled with the cells from the supernatant. Aliquots of 10^6^ cells were fixated with 1% paraformaldehyde (Sigma-Aldrich) and 70% ethanol (Carl Roth) and stored at −20°C. Double staining for infection status and apoptosis was performed using a fluorescein isothiocyanate (FITC)-labeled anti-NP mAb (AbD Serotect) and a terminal deoxynucleotidyl transferase dUTP nick end labeling (TUNEL) assay kit (Roche Diagnostics), respectively. Measurements were collected using an Epics XL flow cytometer (Beckman Coulter). In addition to flow cytometry, the infectious virus titer was measured from the supernatant of T-Flasks using a TCID_50_ assay as described before by Genzel and Reichl [59].

### Real-time RT-qPCR

To extract viral RNAs, cells were washed once with PBS, lysed and scraped from the dish. Lysates were stored at −80°C. RNA was extracted using “INSTANT Virus RNA” (Analytik Jena) according to the manufacturer's instructions and stored at −80°C.

For the real-time RT-qPCR assay, priming strategies for the differentiation of viral RNA species were adapted from Kawakami *et al*. [60]. In brief, polarity specific and tagged primers (Table 2) were used in reverse transcription as follows. 1 µL of RNA extract was mixed with 1 µL primer (1 µM for cRNA and vRNA; 10 µM for mRNA), 1 µL dNTPs (10 mM each) and filled up to 14.5 µL with nuclease-free water. The mixture was incubated at 65°C for 5 min and subsequently cooled to 40°C for mRNA and 55°C for cRNA and vRNA. Afterward, the reaction mixture (4 µL 5×Reaction Buffer, 0.5 µL Maxima H-Minus Reverse Transcriptase (200 U/µL) (Thermo Scientific) and 1 µL nuclease-free water) was added. After incubation at 60°C for 30 min the reaction was terminated at 85°C for 5 min.

**Table 2:** Primer sets for the reverse transcription and real-time RT-qPCR.

Target	Purpose	Primer Name	Sequence (5′-3′)	Position (nt)
mRNA	reverse transcription	Oligo tagdTRT rev	GTAAAACGACGGCCAGTTTTTTTTTTTTTTTTT	polyA tail
	real-time RT-qPCR	Seg 5 Realtime for	GGAAAGTGCAAGACCAGAAGAT	1388–1410
	real-time RT-qPCR	mRNA tagRealtime rev	GTAAAACGACGGCCAGT	tag sequence
cRNA	reverse transcription	Seg 5 tagRT rev	GCTAGCTTCAGCTAGGCATCAGTAGAAACAAGGGTATTTTTCTT	1541–1565
	real-time RT-qPCR	Seg 5 Realtime for	GGAAAGTGCAAGACCAGAAGAT	1388–1410
	real-time RT-qPCR	cRNA tagRealtime rev	GCTAGCTTCAGCTAGGCATC	tag sequence
vRNA	reverse transcription	Seg 5 tagRT for	ATTTAGGTGACACTATAGAAGCGAGTGATTATGAGGGACGGTTGAT	192–215
	real-time RT-qPCR	Seg 5 Realtime rev	CGCACTGGGATGTTCTTC	282–300
	real-time RT-qPCR	vRNA tagRealtime for	ATTTAGGTGACACTATAGAAGCG	tag sequence

Additionally, a 10-fold dilution series of the corresponding RNA reference standards (5⋅10^−7^ to 5 ng) each containing 350 ng cellular total RNA was reverse transcribed. Subsequently, the RT reaction was diluted to a final volume of 100 µL. Concentration of viral RNA was determined in molecules per cell using “Rotor-Gene SYBR Green PCR Kit” (Qiagen) and Rotor-Gene Q real-time PCR cycler (Qiagen). 4 µL of the diluted cDNA were mixed with 1 µL primer set and 5 µL reaction mixture. The cycle conditions of the real-time PCR were 95°C for 5 min followed by 40 cycles of 95°C for 10 sec and 60°C for 20 sec. Finally, a melting curve from 65°C to 90°C was performed. The concentration of viral RNA was calculated based on the RNA reference standards with linear regression (Ct-value against log_10_ of number of molecules). The number of viral RNA molecules (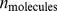) was calculated based on the length of the fragment (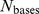 (bp)),
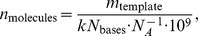
where 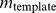 (ng) is the mass of the template, 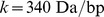 denotes the average mass of one base, and 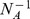 (mol^−1^) corresponds to the Avogadro constant. The number of RNA molecules was then related to the number of cells (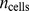 (cells)) to calculate the abundance of viral RNAs per cell (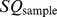 (molecules/cell)). The final result was calculated by
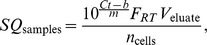
using the coefficient for dilution of RT reaction (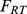) and the volume of RNA eluate (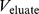 (µL)).

## Supporting Information

Table S1 Initial conditions for the multiscale model. This table lists all non-zero initial conditions that were used in simulations.
(DOC)

Table S2 List of parameters of the intracellular model. This table lists all parameters that were used to simulate the intracellular level of infection along with their units and additional information on their source.
(DOC)

Table S3 List of parameters of the extracellular model. This table lists all parameters that were used to simulate the extracellular level of infection along with their units and additional information on their source.
(DOC)

Table S4 Parameters corresponding to the drug targets in Figure 5 and 7. This table shows which parameters in the model correspond to the drug targets shown in Figure 5 and 7.
(DOC)
