# Supplementary material for: Multiscale Modeling of Influenza A Virus Infection Supports the Development of Direct-Acting Antivirals
Source: PLoS Comput Biol. 2013 Nov 21;9(11):e1003372. doi: 10.1371/journal.pcbi.1003372 (PMC3836700; doi:10.1371/journal.pcbi.1003372)
Supplement: Table S1 — Initial conditions for the multiscale model. This table lists all non-zero initial conditions that were used in simulations. (DOC) [file pcbi.1003372.s001.doc]

**Table S1**. Initial conditions for the multiscale model.

| **Simulation** | **Model** | **Non-zero initial conditions** |
| --- | --- | --- |
| **Figure 1D, 2A** | intracellular | $V^{Ex}\left(0\right)=1.2\text{ virions}$ |
| **Figure 2B** | intracellular | $Vp^{cyt}\left(0\right)=8\text{ molecules}$ |
|  | extracellular | $T\left(0\right)=4.90\times10^{5}\text{ cells}/\text{mL}$ $T_{a}\left(0\right)=9.49\times10^{3}\text{ cells}/\text{mL}$ $V\left(0\right)=6.9\times10^{4}\text{ virions}/\text{mL}$ |
| **Figure 3** | intracellular | $Vp^{cyt}\left(0\right)=8\text{ molecules}$ |
|  | extracellular (MOI 10^-4^) | $T\left(0\right)=5.1\times10^{5}\text{ cells}/\text{mL}$ $T_{a}\left(0\right)=8.9\times10^{3}\text{ cells}/\text{mL}$ $V\left(0\right)=51\text{ virions}/\text{mL}$ |
|  | extracellular (MOI 3) | $T\left(0\right)=5.24\times10^{5}\text{ cells}/\text{mL}$ $T_{a}\left(0\right)=8.06\times10^{3}\text{ cells}/\text{mL}$ $V\left(0\right)=1.57\times10^{6}\text{ virions}/\text{mL}$ |
| **Figure 4, 5, 6** | intracellular | $Vp^{cyt}\left(0\right)=8\text{ molecules}$ |
| **Figure 7** | intracellular | $Vp^{cyt}\left(0\right)=8\text{ molecules}$ |
|  | extracellular | $T\left(0\right)=5.285\times10^{5}\text{ cells}/\text{mL}$ $V\left(0\right)=1\times10^{3}\text{ virions}/\text{mL}$ |
