# Supplementary material for: Multiscale Modeling of Influenza A Virus Infection Supports the Development of Direct-Acting Antivirals
Source: PLoS Comput Biol. 2013 Nov 21;9(11):e1003372. doi: 10.1371/journal.pcbi.1003372 (PMC3836700; doi:10.1371/journal.pcbi.1003372)
Supplement: Table S2 — List of parameters of the intracellular model. This table lists all parameters that were used to simulate the intracellular level of infection along with their units and additional information on their source. (DOC) [file pcbi.1003372.s002.doc]

**Table S1.** List of parameters of the intracellular model.

| **Parameter** | **Description** | **Value** | **Source** |
| --- | --- | --- | --- |
|  | number of high-affinity binding sites | 150 |  |
|  | number of low-affinity binding sites | 1000 |  |
|  | distance between two adjacent ribosomes | 160 |  |
|  | fraction of fusion-competent virions | 0.51 |  |
|  | fraction of M2-encoding mRNAs | 0.02 | based on ratio  of M2 to M1 |
|  | fraction of NEP-encoding mRNAs | 0.125 |  |
|  | attachment to high-affinity binding sites | 8.09×10^-2^ | adjusted to data  in reference |
|  | attachment to low-affinity binding sites | 4.55×10^-4^ | adjusted to data  in reference |
|  | binding of M1 to nuclear vRNPs | 2.43×10^-4^ | model fit in Figure 2 |
|  | binding of NP to  RdRp-RNA complexes | 3.01×10^-4^ |  |
|  | binding of RdRp-complexes to RNA | 1 |  |
|  | degradation of mRNA | 0.33 |  |
|  | degradation of nascent cRNA/vRNA | 36.36 |  |
|  | degradation of RNPs | 0.09 |  |
|  | degradation of RdRp-RNA complexes | 4.25 |  |
|  | endocytosis | 4.8 |  |
|  | equilibrium constant of high-affinity sites | 1.13×10^-2^ |  |
|  | equilibrium constant of low-affinity sites | 8.33×10^-5^ |  |
|  | NEP binding and nuclear export | 1×10^-6^ | adjusted to data  in reference |
|  | fusion with endosomes | 9.56×10^-3^ | model fit in Figure 2 |
|  | nuclear import | 6 |  |
|  | formation of  RdRp- complexes | 1 | assuming rapid complex formation |
|  | virus release/budding | 586 | model fit in Figure 2 |
|  | cRNA synthesis | 5.29 | model fit in Figure 2 |
|  | mRNA synthesis | 8.53×10^5^ | model fit in Figure 2 |
|  | protein synthesis | 64800 |  |
|  | vRNA synthesis | 32.18 | model fit in Figure 2 |
|  | influence of viral components on release | 300 | adjusted to  data in Figure 2 |
|  | length of  segment 1’s mRNA | 2320 |  |
|  | length of  segment 2’s mRNA | 2320 |  |
|  | length of  segment 3’s mRNA | 2211 |  |
|  | length of  segment 4’s mRNA | 1757 |  |
|  | length of  segment 5’s mRNA | 1540 |  |
|  | length of  segment 6’s mRNA | 1392 |  |
|  | length of segment 7’s unspliced mRNA | 1005 |  |
|  | length of segment 8’s unspliced mRNA | 868 |  |
|  | average length  of a vRNA | 1700 | based on  reference |
|  | number of RdRp-complexes in a virion | 45 |  |
|  | number of HA molecules in a virion | 500 |  |
|  | number of NP molecules in a virion | 1000 |  |
|  | number of NA molecules in a virion | 100 |  |
|  | number of M1 molecules in a virion | 3000 |  |
|  | number of M2 molecules in a virion | 40 |  |
|  | number of NEP molecules in a virion | 165 |  |
|  | nucleotides bound by one M1 molecule | 200 |  |
|  | nucleotides bound by one NEP molecule | 1700 | adjusted to data  in reference |
|  | nucleotides bound by one NP molecule | 24 |  |

**Supplementary References**

1. Nunes-Correia I, Ramalho-Santos J, Nir S, de Lima MCP (1999) Interactions of influenza virus with cultured cells: Detailed kinetic modeling of binding and endocytosis. Biochemistry 38: 1095-1101.

2. Arava Y, Wang YL, Storey JD, Liu CL, Brown PO, et al. (2003) Genome-wide analysis of mRNA translation profiles in Saccharomyces cerevisiae. Proceedings of the National Academy of Sciences of the United States of America 100: 3889-3894.

3. Heldt FS, Frensing T, Reichl U (2012) Modeling the Intracellular Dynamics of Influenza Virus Replication To Understand the Control of Viral RNA Synthesis. Journal of Virology 86: 7806-7817.

4. Robb NC, Jackson D, Vreede FT, Fodor E (2010) Splicing of influenza A virus NS1 mRNA is independent of the viral NS1 protein. Journal of General Virology 91: 2331-2340.

5. Amorim MJ, Bruce EA, Read EKC, Foeglein A, Mahen R, et al. (2011) A Rab11-and Microtubule-Dependent Mechanism for Cytoplasmic Transport of Influenza A Virus Viral RNA. Journal of Virology 85: 4143-4156.

6. Babcock HP, Chen C, Zhuang XW (2004) Using single-particle tracking to study nuclear trafficking of viral genes. Biophysical Journal 87: 2749-2758.

7. Spirin AS (1986) Ribosome structure and protein biosynthesis. Menlo Park, CA: Benjamin/Cummings Pub. Co., Advanced Book Program.

8. Lamb RA, Krug RM (2001) Orthomyxoviridae: the viruses and their replication. Fields virology, 4th ed: p.1487-1531. In D. M. Knipe and P. M. Howley (ed.), Fields virology, 4th ed. Lippincott Williams & Wilkins, Philadelphia, Pa.

9. Wakefield L, Brownlee GG (1989) Rna-Binding Properties of Influenza-a Virus Matrix Protein M1. Nucleic Acids Research 17: 8569-8580.

10. Portela A, Digard P (2002) The influenza virus nucleoprotein: a multifunctional RNA-binding protein pivotal to virus replication. Journal of General Virology 83: 723-734.

11. Schulze-Horsel J, Schulze M, Agalaridis G, Genzel Y, Reichl U (2009) Infection dynamics and virus-induced apoptosis in cell culture-based influenza vaccine production-Flow cytometry and mathematical modeling. Vaccine 27: 2712-2722.

12. Beauchemin CAA, McSharry JJ, Drusano GL, Nguyen JT, Went GT, et al. (2008) Modeling amantadine treatment of influenza A virus in vitro. Journal of Theoretical Biology 254: 439-451.
