# Supplementary material for: Multiscale Modeling of Influenza A Virus Infection Supports the Development of Direct-Acting Antivirals
Source: PLoS Comput Biol. 2013 Nov 21;9(11):e1003372. doi: 10.1371/journal.pcbi.1003372 (PMC3836700; doi:10.1371/journal.pcbi.1003372)
Supplement: Table S3 — List of parameters of the extracellular model. This table lists all parameters that were used to simulate the extracellular level of infection along with their units and additional information on their source. (DOC) [file pcbi.1003372.s003.doc]

**Table S1.** List of parameters of the extracellular model.

| **Parameter** | **Description** | **Value** | **Source** |
| --- | --- | --- | --- |
|  | maximum growth rate | 0.03 |  |
|  | number of high-affinity binding sites | 150 |  |
|  | number of low-affinity binding sites | 1000 |  |
|  | ratio of infected cells to fused virions | 1 | model fit in Figure 2 |
|  | increase in apoptosis due to virus infection | 3.28×10^-2^ | model fit in Figure 2 |
|  | apoptosis rate of uninfected cells | 7.35×10^-3^ | model fit in Figure 2 |
|  | attachment to high-affinity binding sites | 3.32×10^-8^ | Adjusted to data  in reference |
|  | attachment to low-affinity binding sites | 1.85×10^-10^ | Adjusted to data  in reference |
|  | degradation/clearance of infectious virus | 0.1 |  |
|  | endocytosis | 4.8 |  |
|  | equilibrium constant of high-affinity sites | 4.48×10^-9^ |  |
|  | equilibrium constant of low-affinity sites | 3.32×10^-11^ |  |
|  | fusion with endosomes | 9.56×10^-3^ | model fit in Figure 2 |
|  | lysis of apoptotic cells | 6.39×10^-2^ | model fit in Figure 2 |
|  | maximum cell concentration | 7×10^5^ | data not shown |

**Supplementary References**

1. Nunes-Correia I, Ramalho-Santos J, Nir S, de Lima MCP (1999) Interactions of influenza virus with cultured cells: Detailed kinetic modeling of binding and endocytosis. Biochemistry 38: 1095-1101.

2. Arava Y, Wang YL, Storey JD, Liu CL, Brown PO, et al. (2003) Genome-wide analysis of mRNA translation profiles in Saccharomyces cerevisiae. Proceedings of the National Academy of Sciences of the United States of America 100: 3889-3894.

3. Heldt FS, Frensing T, Reichl U (2012) Modeling the Intracellular Dynamics of Influenza Virus Replication To Understand the Control of Viral RNA Synthesis. Journal of Virology 86: 7806-7817.

4. Robb NC, Jackson D, Vreede FT, Fodor E (2010) Splicing of influenza A virus NS1 mRNA is independent of the viral NS1 protein. Journal of General Virology 91: 2331-2340.

5. Amorim MJ, Bruce EA, Read EKC, Foeglein A, Mahen R, et al. (2011) A Rab11-and Microtubule-Dependent Mechanism for Cytoplasmic Transport of Influenza A Virus Viral RNA. Journal of Virology 85: 4143-4156.

6. Babcock HP, Chen C, Zhuang XW (2004) Using single-particle tracking to study nuclear trafficking of viral genes. Biophysical Journal 87: 2749-2758.

7. Spirin AS (1986) Ribosome structure and protein biosynthesis. Menlo Park, CA: Benjamin/Cummings Pub. Co., Advanced Book Program.

8. Lamb RA, Krug RM (2001) Orthomyxoviridae: the viruses and their replication. Fields virology, 4th ed: p.1487-1531. In D. M. Knipe and P. M. Howley (ed.), Fields virology, 4th ed. Lippincott Williams & Wilkins, Philadelphia, Pa.

9. Wakefield L, Brownlee GG (1989) Rna-Binding Properties of Influenza-a Virus Matrix Protein M1. Nucleic Acids Research 17: 8569-8580.

10. Portela A, Digard P (2002) The influenza virus nucleoprotein: a multifunctional RNA-binding protein pivotal to virus replication. Journal of General Virology 83: 723-734.

11. Schulze-Horsel J, Schulze M, Agalaridis G, Genzel Y, Reichl U (2009) Infection dynamics and virus-induced apoptosis in cell culture-based influenza vaccine production-Flow cytometry and mathematical modeling. Vaccine 27: 2712-2722.

12. Beauchemin CAA, McSharry JJ, Drusano GL, Nguyen JT, Went GT, et al. (2008) Modeling amantadine treatment of influenza A virus in vitro. Journal of Theoretical Biology 254: 439-451.
