# Supplementary material for: Multiscale Modeling of Influenza A Virus Infection Supports the Development of Direct-Acting Antivirals
Source: PLoS Comput Biol. 2013 Nov 21;9(11):e1003372. doi: 10.1371/journal.pcbi.1003372 (PMC3836700; doi:10.1371/journal.pcbi.1003372)
Supplement: Table S4 — Parameters corresponding to the drug targets in Figure 5 and 7. This table shows which parameters in the model correspond to the drug targets shown in Figure 5 and 7. (DOC) [file pcbi.1003372.s004.doc]

**Table S1.** Parameters corresponding to the drug targets in Figure 5 and 7.

| **Mechanism** | **Parameter** |
| --- | --- |
| assembly of RdRp-complexes | $k^{RdRp}$ |
| binding of M1 to nuclear vRNPs | $k_{M1}^{Bind}$ |
| binding of RdRp to viral RNAs | $k_{RdRp}^{Bind}$ |
| cRNA synthesis | $k_{C}^{Syn}$ |
| encapsidation of viral RNAs by NP | $k_{NP}^{Bind}$ |
| endocytosis of virions | $k^{En}$ |
| fusion with endosomes | $k^{Fus}$ |
| nuclear export of viral genomes | $k^{Exp}$ |
| nuclear import of viral genomes | $k^{Imp}$ |
| viral mRNA synthesis | $k_{M}^{Syn}$ |
| viral protein synthesis | $k_{P}^{Syn}$ |
| virus binding to high-affinity sites | $k_{c.hi}^{Att}$ |
| virus binding to low-affinity sites | $k_{c,lo}^{Att}$ |
| virus assembly/release | $k^{Rel}$ |
| vRNA synthesis | $k_{V}^{Syn}$ |
| splicing of mRNAs for M2 | $F_{Spl7}$ |
| splicing of mRNAs for NEP | $F_{Spl8}$ |
